# A sustained-release drug-delivery system of synthetic prostacyclin agonist, ONO-1301SR: a new reagent to enhance cardiac tissue salvage and/or regeneration in the damaged heart

**DOI:** 10.1007/s10741-015-9477-8

**Published:** 2015-02-24

**Authors:** Satsuki Fukushima, Shigeru Miyagawa, Yoshiki Sakai, Yoshiki Sawa

**Affiliations:** Department of Cardiovascular Surgery, Osaka University Graduate School of Medicine, 2-2 Yamadaoka, Suita, Osaka 565-0871 Japan

**Keywords:** Prostacyclin agonist, Surgical regeneration therapy, Drug delivery system, Translational research, Preclinical studies

## Abstract

Cardiac failure is a major cause of mortality and morbidity worldwide, since the standard treatment for cardiac failure in the clinical practice is chiefly to focus on removal of insults against the heart or minimisation of additional factors to exacerbate cardiac failure, but not on regeneration of the damaged cardiac tissue. A synthetic prostacyclin agonist, ONO-1301, has been developed as a long-acting drug for acute and chronic pathologies related to regional ischaemia, inflammation and/or interstitial fibrosis by pre-clinical studies. In addition, poly-lactic *co*-glycolic acid-polymerised form of ONO-1301, ONO-1301SR, was generated to achieve a further sustained release of this drug into the targeted region. This unique reagent has been shown to act on fibroblasts, vascular smooth muscle cells and endothelial cells in the tissue via the prostaglandin IP receptor to exert paracrinal release of multiple protective factors, such as hepatocyte growth factor, vascular endothelial growth factor or stromal cell-derived factor-1, into the adjacent damaged tissue, which is salvaged and/or regenerated as a result. Our laboratory developed a new surgical approach to treat acute and chronic cardiac failure using a variety of animal models, in which ONO-1301SR is directly placed over the cardiac surface to maximise the therapeutic effects and minimise the systemic complications. This review summarises basic and pre-clinical information of ONO-1301 and ONO-1301SR as a new reagent to enhance tissue salvage and/or regeneration, with a particular focus on the therapeutic effects on acute and chronic cardiac failure and underlying mechanisms, to explore a potential in launching the clinical study.

## Introduction

Cardiac failure is the major cause of mortality and morbidity worldwide [1]. Since cardiac tissue has a limited regenerative capacity, any insults against the heart cause an irreversible myocardial damage dependent upon nature and magnitude of the insult, consequently inducing acute and/or chronic cardiac failure. Current standard to treat cardiac failure is therefore to focus on removal of the insult itself, such as reperfusion of the ischaemic tissue or structural/physiological correction by catheter and/or surgical interventions [2], or minimisation of the additional factors to exacerbate the myocardial damage, such as inhibition of renin–angiotensin–aldosterone system [3, 4] or sympathetic nerve activities [5, 6]. Nonetheless, treatment of cardiac failure is not fully established [7], indicating that it may be therapeutically effective to target on different mechanisms underlying development of cardiac failure, such as enhancement of cardiac tissue salvage and/or regeneration [5].

It has been shown that cardiac-targeted gene transduction of pro-angiogenic/anti-inflammatory factors, such as hepatocyte growth factor (HGF), vascular endothelial growth factor (VEGF) or stromal cell-derived factor (SDF)-1, enhances up-regulation of these factors in the myocardium to contribute to tissue salvage and/or regeneration, consequently inducing functional recovery in an array of the animal models of cardiac failure [8]. Moreover, transplantation of somatic tissue-derived stem/progenitor cells into the heart, such as bone marrow or skeletal muscle-derived cells, persistently induces up-regulation of the pro-angiogenic/anti-inflammatory factors in the myocardium, contributing to functional recovery [9, 10]. It was thus warranted that any treatments targeting intramyocardial up-regulation of pro-angiogenic/anti-inflammatory factors would be promising to enhance myocardial salvage and/or regeneration. In clinical studies, feasibility, safety and therapeutic efficacy of the somatic tissue-derived cell transplantation therapy was established for cardiac failure; however, this treatment has failed to gain a status as the standard in the clinical practice to date for a variety of potential reasons [9, 11]. Firstly, reported magnitude of therapeutic effects is modest, potentially related to limited retention/survival of the transplanted cells and/or limited regeneration capacity of the targeted cardiac tissue [10, 12]. Secondly, additional processes to prepare the cells are required in the routine clinical practice, which would not overweigh the therapeutic benefits expected by the cell transplantation therapy. It is therefore theorised that “the shelf-stored drug” which has similar therapeutic effects to the cell transplantation therapy would be more widely used, potentially accepted as the standard, regeneration-based therapy for cardiac disease in the clinical practice [13].

**Fig. 1:**
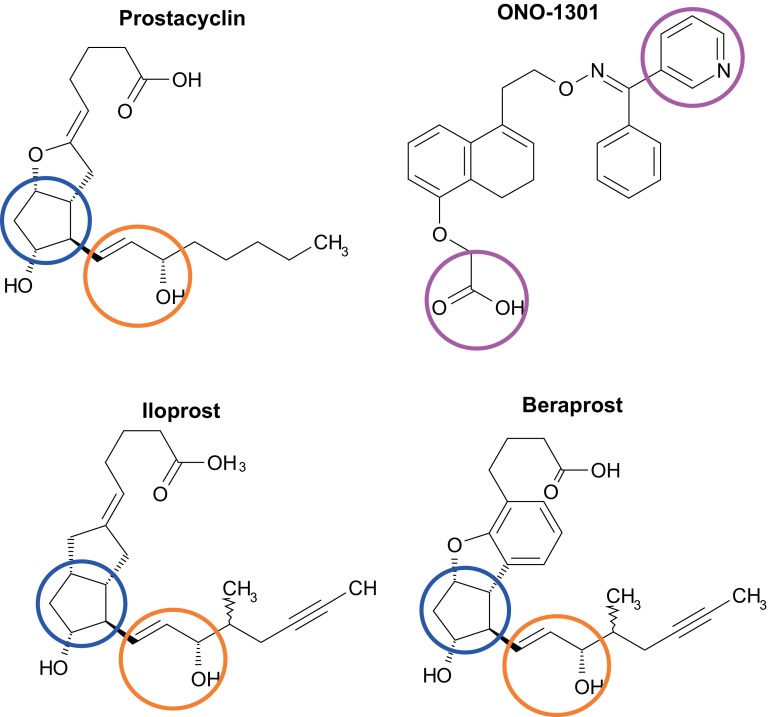
Prostacyclin has the prostanoid structure including a five-membered ring (indicated as *blue* *circle*) and an allylic alcohol (indicated as *orange* *circle*), which are rapidly metabolised in vivo. In contrast, a synthetic selective agonist of prostacyclin, ONO-1301, lacks the prostanoid structure, while this reagent has the structure having a thromboxane A2 inhibitory activity (indicated as *purple circle*). Other selective prostacyclin agonists, such as iloprost or beraprost, have the prostanoid structure (indicated as *blue* and *orange circles*) without thromboxane A2 inhibitory activity

Prostaglandins and their derivatives are endogenous autacoids produced by cyclooxygenase-related arachidonic acid cascade, contributing to vasodilatation [14], inhibition of platelet aggregation or anti-inflammation [15] in response against local tissue damage. Of a variety of synthetic agonists or antagonists of this cascade that were developed as a drug, ONO-1301 (7,8-dihydro-5-[(*E*)-[[a-(3-pyridyl)-benzylidene]aminooxy]ethyl]-1-naphthyloxy]acetic acid) was synthesised as a small molecular weight compound having prostacyclin IP receptor agonistic and thromboxane A2 synthase inhibitory activities (Fig. 1) [16–19]. ONO-1301 was initially developed as an anti-platelet drug; however, a number of the basic studies including those from our laboratory documented that a very small dose of ONO-1301 activates endothelial cells, vascular smooth muscle cells and fibroblasts to release multiple pro-angiogenic/anti-inflammatory factors from their cytoplasm [16, 20–23]. In addition, ONO-1301 has several theoretical advantages as a drug over other synthetic prostaglandin agonists, such as beraprost, epoprostenol, iloprost or treprostinil, since ONO-1301 has been shown to exert long-lasting prostacyclin activities [16, 24, 25]. Moreover, poly-lactic *co*-glycolic acid (PLGA)-polymerised form of ONO-1301, ONO-1301SR, was developed to achieve a sustained-release ONO-1301-delivery-system [16, 25]. This review summarises information and knowledge regarding to ONO-1301 and ONO-1301SR as a tissue regeneration-based drug for cardiac disease and proposes prospect of ONO-1301 for new drug in chronic pathologies.

## Pharmacological activity of ONO-1301 and development of ONO-1301SR

ONO-1301 is prostacyclin IP receptor agonist with a thromboxane A2 synthase inhibitory activity. ONO-1301SR is a PLGA-polymerised ONO-1301 to achieve a sustained-release system [25]. These products have been shown to have therapeutic effects on a variety of cardiac pathologies via cytokines-induced salvage and/or regeneration of damaged cardiac tissue. This section documents characteristics and pharmacological activity of ONO-1301 and ONO-1301SR in vitro. In addition, other prostaglandin agonists under development are discussed in comparison with ONO-1301.

### Characteristics of ONO-1301

ONO-1301 is a synthetic prostacyclin IP receptor agonist lacking the typical prostanoid structures, including a five-membered ring and allylic alcohol, which are rapidly metabolised by 15-hydroxyprostaglandin dehydrogenase in vivo (Fig. 1) [16]. It is thus indicated that ONO-1301 is a chemically stable structured prostacyclin agonist. In addition, ONO-1301 has a 3-pyridine radical to exert a thromboxane A2 synthase inhibitory activity, which induces an intrinsic prostaglandin I2 synthesis-promoting effect to augment the IP receptor agonistic activity [16]. Therefore, this unique structure of ONO-1301 has been shown to produce a long-lasting prostacyclin activity with little drug resistance compared to other prostacyclin agonists used in the clinical settings [24], proposing the advantage of this new drug for acute and chronic pathologies that are related to ischaemia, inflammation and/or fibrosis. In addition, it was reported that ONO-1301 is inactivated by oxidation in the liver within 3–4 h [24], indicating a wide utility of this product as a drug in the clinical settings.

### Pharmacological activity of ONO-1301

It has been shown that ONO-1301 agonises the IP receptor expressed in a variety of the cells, such as fibroblast, vascular smooth muscle cell or endothelial cell, to up-regulate expression of multiple factors, such as VEGF, HGF or SDF-1, in vitro [16]. The effects of ONO-1301 as a cytokine inducer were shown to be mediated at least in part by elevation of intracellular cyclic adenosine monophosphate (cAMP) [16, 26]. In addition, extracellularly released factors by ONO-1301 have been shown to enhance a tube-like formation of human umbilical vein endothelial cells (HUVECs) co-cultured with normal human dermal fibroblasts (NHDF) in vitro [27], indicating a pro-angiogenic property of ONO-1301. In addition, it was reported that NHDF stimulated by ONO-1301 enhanced migration of bone marrow (BM)-derived cells mediated by extracellularly released SDF-1, in vitro [20], suggesting that ONO-1301 may have an effect to enhance migration of circulating BM cells into the targeted territory contributing to BM cell-mediated tissue salvage and/or regeneration.

### Development of ONO-1301SR to establish a sustained-release drug-delivery system

**Fig. 2:**
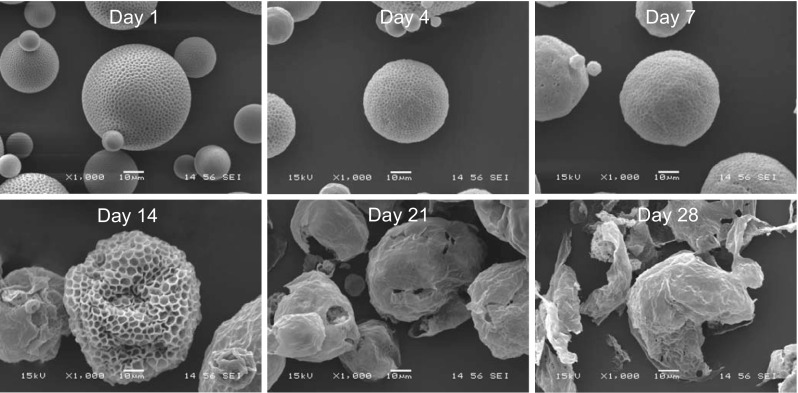
Representative electron micrographic images of ONO-1301SR, which is a PLGA-polymerised form of ONO-1301, after production at 37 °C in vitro. Structure of the microspheres is gradually degraded over 28 days

While ONO-1301 has been shown to have a long-lasting prostacyclin agonistic effect compared to the other prostacyclin agonists, it would be further useful and beneficial to develop a sustained-release drug-delivery system of ONO-1301 to achieve a further prolonged prostacyclin agonistic effects on the targeted territory of acute and chronic pathologies. For this purpose, ONO-1301 was polymerised with PLGA microspheres that are proven to be biocompatible and biodegradable, used as controlled delivery system for proteins or drugs in clinical settings [16, 25]. As a result, this ONO-1301SR product was shown to be hydrolysed at the site of administration to linearly release ONO-1301 into the adjacent tissue with a modest initial burst (Fig. 2). In addition, duration of ONO-1301 release can be adjusted by modifying the molecular weight of PLGA, the lactic/glycolic acids ratio or the particle size to achieve optimum effects, depending upon the targeted pathology or drug delivery mode [25].

### Other prostaglandin agonists under development

Agonists of prostagrandins are theoretically therapeutic for acute and chronic pathologies associated with tissue ischaemia, inflammation and/or interstitial fibrosis. Xiao et al. [28] reported in 2004 that prostaglandin EP4 receptor agonist, ONO-4819, was effective in attenuating myocardial ischaemia–reperfusion injury via elevation of intracellular cAMP concentration in noncardiomyocytes. In addition, this product was shown to have a positive effect on bone regeneration [29–39] or nerve root angiogenesis [40], and have a protective effect against acute liver injury [41], skin injury [42] or renal tubulointerstitial fibrosis [43]. Of note, ONO-4819 is under the clinical study for treating medically refractory ulcerative colitis [44], though the result has not been reported. Another EP4 receptor agonist, EP4RAG, has been shown to have a protective effect against ischaemia–reperfusion myocardial injury [45], cardiac allograft transplantation-related inflammation [46] or experimental autoimmune myocarditis [47]. Despite several similar products to ONO-1301, it appears that sustained-release form of prostagrandin agonist has been developed only in ONO-1301 to date.

## Evidence of cardiac tissue salvage/regeneration by ONO-1301SR

Therapeutic effects of ONO-1301SR have been tested on a variety of cardiac pathologies, such as acute and chronic myocardial infarction (MI), cardiomyopathy, cardiac allograft disease post-transplantation or myocarditis. As a result, ONO-1301SR treatment showed positive effects on these pathologies by different mechanisms from other existing treatments that are used in the clinical settings, indicating that ONO-1301SR is a potential new drug for a variety of cardiac diseases. This section summarises previous reports that document effects of ONO-1301 or ONO-1301SR on each cardiac pathologies.

### Effects of ONO-1301SR on acute MI

Ischaemic insult against the heart by limiting coronary perfusion rapidly induces intracellular lactic acidosis in the cardiac myocytes, which leads to reduction of cellular contractility and subsequent necrotic cell death, consequently generating a state of “acute MI” [48, 49]. An array of debris from the dead cells or “danger signals” from cells that confront with the ischaemia activate inflammatory reactions, including accumulation of circulating cells or activation of residential cells in the cardiac tissue that consequently determines an area of “infarct region,” where cardiac myocytes were lysed and replaced by fibrous components [50, 51]. In addition, border area between the infarct area and the area with sufficient blood supply confronts with persistent ischaemia that progressively widen the infarct region [51].

Treatment for the acute MI is therefore reperfusion of the ischaemic area to supply sufficient blood flow into the tissue [52]. It is, however, known that early reperfusion induces intracellular calcium overload, overproduction of superoxides and their derivatives and mitochondrial permeability transition pore opening in the cardiac myocytes, which consequently yields rapid cell death that often causes lethal ventricular arrhythmia, and importantly exacerbates inflammatory response in the reperfused area [53, 54]. Despite a number of attempts, additional treatments that effectively reduce ischaemia–reperfusion cardiac injury have not been developed [53]. Among the agents that activate myocyte receptors, such as adenosine [55], bradykinin [56], opioids [57], glucagon-like peptide 1 [58], atrial natriuretic peptide (ANP) [59], erythropoietin [60] or insulin [61], intravenous infusion of adenosine and ANP showed positive, but not substantial, additional therapeutic effects to direct percutaneous coronary intervention for acute MI. In addition, effects of the agents that act intracellularly, such as volatile anaesthetics [62], nitrates [63], atorvastatin [64], delcasertib [65], nicorandil [59] or cyclosporine [66], have not been proven by large randomised studies. There are a number of other potential agents that were or were not tested by large-scale human studies, such as phosphodiesterase-5 inhibitors [67], superoxide dismutase [68] or neutralising antibodies against adhesion molecules such as P-selectin, intercellular adhesion molecule-1 [69]. Importantly, these treatments are targeted to effect on a single cellular and/or molecular process among a variety of complicated dynamic processes related to ischaemic-reperfusion cardiac injury, potentially limiting the overall therapeutic effects. In addition, delivery method of the agents needs to be optimised depending upon the underlying therapeutic mechanisms [68, 69].

In contrast, it has been shown that administration of ONO-1301SR directly activates endothelial cells and vascular smooth muscle cells through the IP receptor, to induce paracrinal release of protective factors, such as HGF, VEGF or SDF-1, into the damaged cardiac tissue. Nakamura et al. [27] first reported therapeutic effects of ONO-1301SR on acute MI in 2007. They directly injected ONO-1301SR into the myocardium that was subjected to ischaemia by left coronary artery ligation in mice. As a result, LV enlargement post-MI was ameliorated and survival was improved by ONO-1301SR treatment, in association with intramyocardially up-regulated HGF and VEGF. They concluded that angiogenesis by ONO-1301SR-induced up-regulation of multiple cytokines is the key therapeutic mechanisms of this treatment for acute MI [27]. In addition, the same team reported the angiogenesis-related positive effects of ONO-1301SR on acute MI with reperfusion using a rat model in 2012 [70]. Furthermore, our group reported that ONO-1301SR treatment enhances recruitment of bone marrow-derived cells into the ischaemic myocardium via enhanced SDF-1/C-X-C chemokine receptor type 4 interaction in a murine acute MI model [20]. It was thus concluded that accumulation of bone marrow-derived cells by ONO-1301SR treatment is an alternative therapeutic mechanisms of this treatment, though role of the accumulated cells needs to be clarified. Noticeably, our group delivered ONO-1301SR into the heart in an atelocollagen-based sheet form [20], since it was considered that direct injection of ONO-1301SR into the myocardium may induce myocardial injury.

All of these reports suggest mechanisms of cardiac salvage and/or regeneration in the ONO-1301SR treatment for acute MI, such as angiogenesis by up-regulation of multiple pro-angiogenic cytokines or recruitment of bone marrow-derived cells. However, further basic studies are necessary to thoroughly clarify the therapeutic mechanisms of this treatment for acute MI. Moreover, these reports indicate the therapeutic potential of ONO-1301SR for treating acute MI in clinical settings, whereas delivery method and dose of ONO-1301SR need to be optimised in basic studies by the good laboratory practice (GLP) standard.

### Effects of ONO-1301SR on chronically failing heart

Chronic cardiac failure is a result of previous or continuous insult against the heart, such as ischaemia, valvular pathologies or genetic abnormalities. In this state, pressure and/or volume overload in the heart continuously activates a variety of cellular and molecular processes to remodel ventricular structure, by which pressure and/or volume overload is further exacerbated, to generate the viscous cycle, “left ventricular (LV) remodelling” [5]. In addition, humoral, hormonal and/or sympathetic nerve activities further exacerbates pressure and/or volume overload to affect progression of the LV remodelling.

Existing surgical treatments directly target pressure and/or volume overloading by intervening valvular pathologies or dilated ventricle, while existing medical treatments target hormonal and/or sympathetic nerve activities. On the other hand, treatments targeting responsible cellular and molecular processes for LV remodelling are under development as represented by cell transplantation therapy [9, 10, 12]. It has been shown that transplantation of somatic tissue-derived stem/progenitor cells, such as bone marrow-derived cells or skeletal muscle-derived cells, into the chronically failing heart enhanced native regeneration capacity by inducing constitutive expression of pro-angiogenic factors or anti-fibrotic factors, consequently to reverse LV remodelling, as reported by an array of pre-clinical studies [9, 10, 12]. Treatment by ONO-1301SR was also reported to induce similar therapeutic mechanisms to the cell transplantation therapy in chronic failing heart in pre-clinical studies as follows.

Iwata et al. [71] generated a chronic MI model in swine by placing the ameroid constrictor in the left circumflex artery (LCX) to induce MI. Four weeks later, they performed direct epicardial injection of ONO-1301SR into the infarct border area under LV electromechanical mapping guidance using a transcatheter system. As a result, ONO-1301SR treatment enhanced collateral growth in relation to increased number of the capillaries, attenuated collagen fraction in the myocardial interstitium and reduced LV volume, indicating that this treatment reversed the LV remodelling. They discussed that intramyocardially delivered ONO-1301SR directly acted on the residential fibroblasts to induce up-regulation of cardiotherapeutic factors such as VEGF or HGF, which in turn activated the regeneration process in the chronic MI heart [71].

**Fig. 3:**
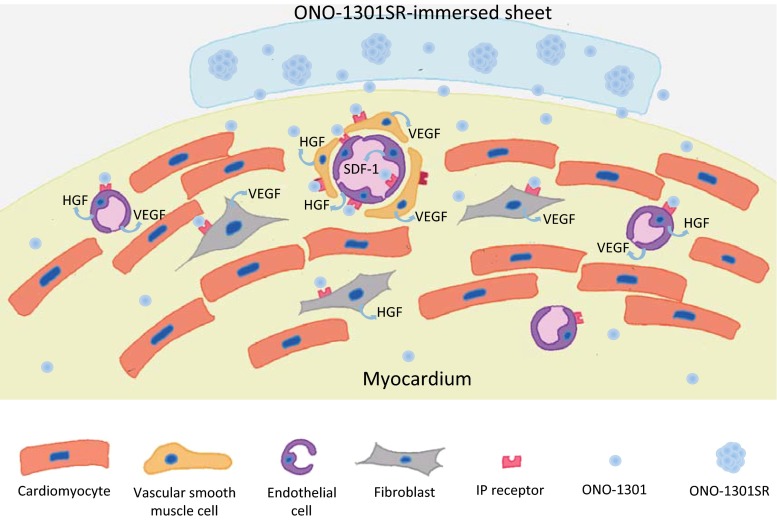
Schematic representation of proposed mechanisms underlying ONO-1301SR-immersed sheet placement therapy for treating damaged cardiac tissue. ONO-1301 is linearly released from the ONO-1301SR by hydrolysis and infiltrated into the cardiac tissue. IP receptor-expressing cardiac cells, such as vascular smooth muscle cells, fibroblast and endothelial cells, are activated by ligation of ONO-1301 to paracrinally release protective factors, such as VEGF, HGF or SDF-1

Our group generated a chronic MI model in canine by permanently ligating left coronary artery [22]. Subsequently, ONO-1301SR-immersed atelocollagen sheet was placed over the LV surface of this model. ONO-1301SR treatment induced functional recovery compared to sham treatment as assessed by standard and speckle-tracking echocardiography and cardiac catheterisation studies, in association with up-regulated HGF, VEGF or SDF-1. Importantly, this study showed increased myocardial blood flow by ONO-1301SR treatment as assessed by ^13^N-ammonia positron emission tomography study [22], indicating that pro-angiogenic effects of ONO-1301SR augment myocardial blood flow to induce functional recovery in ischaemic cardiomyopathy (Fig. 3).

Effects of ONO-1301SR on dilated cardiomyopathy were tested by Hirata’s group and our group. Hirata et al. [72] subcutaneously injected ONO-1301SR into the hamsters having genetically determined dilated cardiomyopathy. As a result, ONO-1301SR-treated hamsters showed a preserved cardiac function in relation to reduced fibrous components and increased capillary network in the myocardial interstitium, suggesting a therapeutic potential of systemic delivery of ONO-1301SR into the dilated cardiomyopathy-related chronic cardiac failure [72].

In contrast, our group used the rapid-pacing-induced canine model [23] and the delta-sarcoglycan-deficient hamster model [21] that was a different model from that of Hirata’s group. Our group directly delivered into the heart in order to maximise the therapeutic effects of this reagent and minimise systemic complications. Firstly, ONO-1301SR was intramyocardially injected in rapid LV-paced canines with their LV ejection fraction being <40 % [23]. As a result, global and regional LV functions were recovered in 4 weeks after the treatment, in association with increased microvessel number, decreased myocyte diameter and decreased fibrous component in the LV myocardium [23]. However, the direct intramyocardial injection of ONO-1301SR used in this report was concerned by inconsistent delivery of the reagent and injection-related myocardial injury.

Therefore, in the subsequent study, ONO-1301SR immersed into the atelocollagen sheet was simply placed on the LV surface of hamster model of dilated cardiomyopathy [21]. This delivery method would achieve a consistent delivery of the reagent into the heart and minimum injury to the myocardium, whereas the myocardial territory that is affected by ONO-1301SR might be theoretically limited. As a result, myocardial vascular network was globally and homogeneously better developed in the ONO-1301SR-treated hamsters with a substantial survival benefit in association with up-regulated cardiotherapeutic factors such as VEGF, HGF or SDF-1 [21]. Importantly, this study showed a consistently heart-dominant elevation of ONO-1301 concentration until 4 weeks after the ONO-1301SR administration [21]. It was thus indicated that placement of ONO-1301SR-immersed collagen sheet over the LV surface act on the entire LV contributing to global functional recovery. These two studies proved the concept that local delivery of ONO-1301SR into the heart contributes to functional recovery and survival benefit in dilated cardiomyopathy-related chronic cardiac failure (Fig. 3).

### Effects of ONO-1301SR on other cardiac pathologies

ONO-1301SR was thus shown to have anti-inflammatory, pro-angiogenic and anti-fibrotic effects on acute and chronic cardiac pathologies via up-regulation of a variety of cardiotherapeutic cytokines and chemokines. Furthermore, positive effects of ONO-1301SR were shown in other cardiac pathologies such as cardiac graft disease post-transplantation [73] or autoimmune myocarditis.

Suzuki et al. [73] subcutaneously injected ONO-1301SR into the mice that were subjected to heterotopic cardiac allograft transplantation in the aim to test the effects of ONO-1301SR on acute and chronic graft-host disease. This treatment was effective in chronic rejection as shown in the reduced myocardial fibrosis in a class II mismatch combination, but not effective in acute rejection in a full allomismatch combination, suggesting that ONO-1301SR might be a potential new drug for chronic rejection post-cardiac allograft transplantation. Hirata et al. [74] reported that daily intake of ONO-1301 (not PLGA-polymerised SR form, but bulk substance) suppressed a progression of LV remodelling chiefly via up-regulation of HGF in a rat autoimmune myocarditis model. It was indicated that HGF plays a critical role in LV remodelling in this model and that ONO-1301 may be an ideal inducer of HGF in the myocardium. Further studies are warranted to prove the positive effects of ONO-1301 on other cardiac pathologies related to acute/chronic inflammation, microvascular dysfunction or fibrous accumulation in the myocardium, such as hypertensive cardiac disease.

## Towards clinical studies of ONO-1301SR for treating cardiac disease

Although positive effects of ONO-1301SR treatment were proven on a variety of cardiac pathologies, such as acute MI, idiopathic dilated cardiomyopathy, ischaemic cardiomyopathy, cardiac allograft disease post-transplantation or fulminant myocarditis, it is a key of success of this treatment to optimise delivery method of ONO-1301 for each target pathology in clinical study. This section discusses suitable target pathology and delivery method of this reagent, and studies necessary for the first-in-human study. In addition, potential methods to enhance the therapeutic effects of ONO-1301 are discussed in the prospect of clinical application.

### Pathology-specific ONO-1301SR delivery for the clinical study

It has been shown that both systemic and local delivery of the ONO-1301SR potentially contributes to the therapeutic benefits for acute and chronic cardiac disease. However, optimal mode of the delivery in the clinical settings would be dependent upon the pathology and, most importantly, the standard treatment for the pathology in the routine clinical practice.

Since the standard treatment for the acute MI is the prompt reperfusion of the occluded coronary arteries by percutaneous transcatheter approach, direct intramyocardial injection of ONO-1301SR by transcatheter approach at the time of reperfusion may be ideal, although further basic studies using a large animal model are necessary. In addition, subcutaneous ONO-1301SR injection or oral intake of ONO-1301 would be optional mode of the ONO-1301SR delivery as an additional treatment for acute MI to the standard reperfusion therapy.

The standard treatment for ischaemic and non-ischaemic dilated cardiomyopathy is the intensive combination of medical and interventional treatments. Addition of subcutaneous ONO-1301SR injection or oral ONO-1301 intake to the optimal medical management would augment the therapeutic effects of the standard medical treatment. Placement of ONO-1301SR over the cardiac surface, which has been intensively developed by our laboratory, may be added at the time of coronary artery bypass grafting and/or mitral valve surgery for ischaemic and non-ischaemic dilated cardiomyopathy. Addition of the ONO-1301SR placement therapy at the time of ventricular assist device implantation surgery might be effective in enhancing functional recovery to achieve “bridge-to-recovery” for dilated cardiomyopathy or fulminant myocarditis.

Head-to-head comparison study for the therapeutic effects between placement over the heart and subcutaneous injection of ONO-1301SR has not been reported. However, it may be theorised that enhanced effects will be achieved by the local placement which maximise ONO-1301 delivery into the targeted area with minimal systemic effects, since paracrinal actions of the effector cells are augmented in positive relation to the magnitude of the ONO-1301 stimuli [20, 21, 23].

### Studies necessary for the clinical study

Once the target pathology and the delivery mode of the ONO-1301SR treatment were decided, dose-optimisation study ideally using a large animal model is necessary to launch the clinical study. Degree of therapeutic effects and systemic complications such as hypotension, bleeding or diarrhoea, in addition to plasma and cardiac ONO-1301 level, need to be investigated depending upon the dose of ONO-1301SR. Moreover, toxicity test in the GLP standard is necessary by using a large animal model over 3 months, since it was shown that ONO-1301 is extinguished from the body by 1 month [21].

### Enhancing positive effects of ONO-1301SR treatment on cardiac pathologies

Therapeutic mechanisms of ONO-1301SR were to induce constitutive up-regulation of a variety of protective factors, such as VEGF, HGF or SDF-1, which are shared by somatic tissue-derived stem/progenitor cell transplantation therapy. One may be concerned by durability of the therapeutic efficacy, since all administered ONO-1301 as a SR form is inactivated by 4 weeks. Although newly formed vasculatures by 4 weeks might remain to contribute to the myocardial blood flow and thus the functional recovery despite extinction of paracrinal stimuli, as seen in the tissue-derived stem/progenitor cell-based therapy [75], additional concomitant treatments may augment the positive effects and prolong its durability. One approach would be combination with the treatments that contribute to cardiac function by a different mechanism from ONO-1301SR, while the other approach may be combination with treatments whose mechanisms are similar to ONO-1301SR.

Our group developed hybrid therapy by combination of ONO-1301SR delivery and cardiac support mesh net device placement [22]. It has been shown that placement of cardiac support net over the ventricles mechanically reduces diastolic LV wall stress to inhibit progression of the LV remodelling, whereas clinical studies of cardiac support net for treating chronically failing heart failed to show survival benefits despite positive effects on the LV volume [76]. This inconsistent result of cardiac support net device would be explained by a lack of biological effects in this treatment. In contrast, ONO-1301SR contributes to recovery of cardiac function by the biological effects, not by mechanical effects. It was therefore theorised that combination of ONO-1301SR and cardiac support net placement would augment the therapeutic effects of either treatment. To test this hypothesis, our laboratory developed a hybrid device consisting of biodegradable polyglycolic acid-based cardiac support net and ONO-1301SR-immersed atelocollagen sheet for treating a canine chronic MI model [22]. As a result, the hybrid device elicited a greater reversal of the MI-inducing LV remodelling than either single treatment, indicating the potential of this device for chronic cardiac failure [22].

Transplantation of somatic tissue-derived stem/progenitor cells has been shown to yield a functional recovery of the failing heart via a similar mechanism to the ONO-1301SR treatment, though therapeutic effects of the cell transplantation therapy are reportedly limited by poor initial retention and long-term survival of the transplanted cells [75, 77, 78]. One may claim that head-to-head comparison study in the therapeutic effects of the two treatments would be clinically important. This pre-clinical study may not be, however, justified by a large number of model animals used to gain statistical significance, since previous studies showed that both treatments improved ejection fraction by 5–10 % [22, 79]. Rather, addition of the ONO-1301SR placement therapy to the cell transplantation therapy may prolong the regenerative effects for the cardiac tissue, depending upon expression of IP receptor and subsequent intracellular signalling in the transplanted cells. Further studies are necessary to test this hypothesis.

Omentum is an abdominal organ, mobilised to be attached to the abdominal organ/tissue in response to the tissue damage/injury. Multiple pro-angiogenic factors are known to be abundantly expressed in the omentum, contributing to regeneration and/or healing of the damaged/injured tissue/organ. This unique character of the omentum was applied to develop a treatment for cardiac ischaemic disease by mobilising to the cardiac surface in a pedicle fashion [75, 80]. As a result, angiogenesis was induced in the ischaemic/infarct territory of the cardiac tissue. Of note, it was reported that omentum covering over the chronic MI-heart with local sustained-delivery of basic fibroblast growth factor (bFGF), but not without bFGF, induced a new vascular network formation between the pedicle omentum and the heart [75]. It is thus theorised that the omentum covering with local delivery of ONO-1301SR might be effective in augmenting regional blood flow in the ischaemic cardiac tissue via formation of new vascular networks in the heart.

## ONO-1301/ONO-1301SR treatment for extracardiac pathologies

ONO-1301 is theoretically effective in treating any acute and chronic pathologies for which dysfunction of microvasculature or accumulation of fibrous components in the tissue/organ is responsible, as shown in the studies for cardiac pathologies. In fact, use of this reagent was reported to be effective in pulmonary arterial hypertension (PAH), pulmonary fibrosis or chronic kidney disease. Moreover, the effects of ONO-1301SR as a potent protective cytokines-inducer might be applied to other pathologies, such as cerebral, liver or pancreatic pathologies. This section summarises previous reports and potential target of ONO-1301SR treatment for extracardiac pathologies. In addition, this section discusses a potential of ONO-1301SR in combination with medical devices, to accumulate further knowledge and information regarding this unique product, exploring further applications.

### ONO-1301SR treatment for lung disease

Since several prostagrandin agonists are the standard treatment of primary and secondary PAH in the clinical practice [81, 82], an ideal target pathology of ONO-1301 and/or ONO-1301SR treatment might be PAH or associated lung diseases. In fact, Kataoka et al. reported in 2006 that repeated subcutaneous administration of ONO-1301 attenuated monocrotaline-induced PAH in rats via the long-lasting cAMP stimulation and thromboxane synthase inhibition [83, 84]. Subsequently, Obata et al. [85] reported in 2007 that a single subcutaneous injection of ONO-1301SR resulted in attenuated pulmonary arterial pressure, at least in part, through inhibition of vascular smooth muscle cell proliferation in a rat monocrotaline-induced PAH model. The same group reported in 2013 that oral administration of ONO-1301 was therapeutic in monocrotaline-induced PAH rats [86]. Moreover, Murakami et al. [87] reported in 2006 that repeated subcutaneous administration of ONO-1301 attenuated bleomycin-induced pulmonary fibrosis in mice.

Hayashi et al. [88] reported in 2010 that administration of ONO-1301 was more therapeutic for ovalbumin-induced asthma model in mice than beraprost. In addition, Kimura et al. [89] tested the hypothesis that ONO-1301SR treatment is effective in suppressing hyperresponsiveness, allergic inflammation and remodelling of the airway. As a result, they proved the anti-inflammatory and the reverse remodelling effects of ONO-1301SR on chronic house dust mite-induced asthma model in mice.

These results might warrant a potential of ONO-1301SR treatment for PAH or asthma, of which chronic inflammation is involved in the development of the pathologies, in clinical practice. Further basic studies should be focused on optimisation of the dose of ONO-1301SR or the administration mode of ONO-1301SR, such as a single subcutaneous injection, intermittent injections, or oral intake. It may be proposed that intravenous injection of ONO-1301SR would induce entrapment of the product in the pulmonary arterioles or capillaries to achieve sustained release of the ONO-1301, although intravenous injection may carry a substantial risk of pulmonary embolism that further exacerbates PAH or associated lung pathologies.

### ONO-1301SR treatment for kidney diseases

Progression of chronic kidney disease is known to be regulated by chronic inflammatory and fibrotic process in the tubulointerstitium of the kidney. Anti-inflammatory effects of ONO-1301 on nephritis was first reported by Hayashi et al. in 1997 [90]. Subsequently, Yamasaki et al. [91] reported that repeated injections of ONO1301SR were effective in reducing renal fibrosis in diabetic nephropathy rat model. In addition, Nasu et al. [92] reported in 2012 that a single subcutaneous injection of ONO-1301SR into the mice that were subjected to unilateral ureteral obstruction yielded a suppression of interstitial fibrous components of the obstructed kidney partly via inhibition of transforming growth factor (TGF)-β, suggesting a potential of ONO-1301SR for the chronic kidney disease, though further studies to prove the safety, efficacy and further mechanisms underlie this treatment.

### ONO-1301SR treatment for other organ pathologies

Although standard treatment of cerebral ischaemia is early reperfusion, additional medical treatments that ameliorate ischaemia–reperfusion injury would further improve the outcome of the intervention. Hazekawa et al. [93] reported in 2012 that a single subcutaneous injection of ONO-1301SR into the rats that were subjected to repeated induction of cerebral ischaemia yielded a short-term functional and histopathological recovery. The same groups reported in 2012 that repeated ONO-1301SR administration reduced ischaemic damage of rats that were subjected to middle cerebral artery occlusion [94].

Acute liver injury is a life-threatening disorder, initiated by a burst inflammation, followed by a complex inflammatory process. Since prompt treatment is known to improve the outcome of this pathology, new “shelf-stored drug” has been long sought. Xu et al. [95] reported in 2011 that intermittent oral administration of ONO-1301, not SR product, ameliorated CCl4-induced acute hepatic injury in mice partly via up-regulation of HGF. The same group reported in 2013 that ONO-1301SR was effective in treating CCl4-induced inflammatory chronic liver fibrosis in mice [96]. Inflammation plays a key role in clinical and pathological progression of chronic pancreatitis. Niina et al. [97] reported in 2014 that ONO-1301SR inhibited monocyte activity to suppress pancreatic fibrosis. These reports indicate that ONO-1301SR may be therapeutically effective for acute and chronic pathologies related to ischaemia, inflammation and/or fibrosis in multiple organs.

### A potential of ONO-1301SR in combination with medical devices

Of the implantable medical devices that have been recently developed, vascular stent has been widely used as the standard treatment for atherosclerotic arterial stenosis or aortic aneurysm [98]. In particular, stent graft implantation of aortic aneurysm has improved clinical outcomes of this pathology, though complications related to a poor attachment of the stent and the native aortic wall have not been fully resolved [99]. Since ONO-1301 has effects on tissue healing and/or regeneration, it is hypothesised that local delivery of ONO-1301 might strengthen the attachment between the stent graft and the native aortic wall. To test this hypothesis, our laboratory developed an aortic stent graft that was coated with ONO-1301SR and implanted in the thoracic aorta of canines [100]. As a result, the attachment was physiologically and histopathologically strengthened. This concept may be applicable to the transcatheter aortic valve replacement, in which aortic valve incompetence caused by suboptimal attachment of the prosthesis and the native aortic annulus yields a negative impact of this treatment [101].

## Conclusions

Feasibility, safety and therapeutic efficacy of a synthetic prostacyclin agonist, ONO-1301, and a sustained-release form of ONO-1301, ONO-1301SR, have been tested in a variety of acute and chronic pathologies related to ischaemia, inflammation and fibrosis of multiple organs including the heart as pre-clinical studies. Major mechanisms underlying the therapeutic effects were consistently to induce release of multiple protective cytokines including HGF, VEGF or SDF-1 from targeted fibroblasts, vascular smooth muscle cells or endothelial cells, which enhance salvage and/or regeneration of the damaged tissue, including the heart.

Both acute and chronic cardiac failure related to ischaemic or non-ischaemic aetiologies would be a target of this novel treatment. Since direct placement of ONO-1301SR over the cardiac surface was suggested to be an optimal treatment for chronic cardiac failure using this product, launching the clinical study of this treatment is warranted. In addition, oral administration of ONO-1301 would be a potential drug for chronic cardiac failure, though further pre-clinical studies are needed in the GLP standard.

## Abbreviations

ANP: Atrial natriuretic peptide
bFGF: Basic fibroblast growth factor
BM: Bone marrow
cAMP: Cyclic adenosine monophostate
GLP: Good laboratory practice
HGF: Hepatocyte growth factor
HUVEC: Human umbilical vein endothelial cell
LV: Left ventricle
MI: Myocardial infarction
NHDF: Normal human dermal fibroblast
PAH: Pulmonary arterial hypertension
PLGA: Poly-lactic *co*-glycolic acid
SDF: Stromal cell-derived factor
TGF: Transforming growth factor
VEGF: Vascular endothelial growth factor

## References

[1] Felker GM, Shaw LK, O’Connor CM (2002). A standardized definition of ischemic cardiomyopathy for use in clinical research. J Am Coll Cardiol.

[2] Abraham WT, Smith SA (2013). Devices in the management of advanced, chronic heart failure. Nat Rev Cardiol.

[3] Jiang F, Yang J, Zhang Y (2014). Angiotensin-converting enzyme 2 and angiotensin 1–7: novel therapeutic targets. Nat Rev Cardiol.

[4] Lang CC, Struthers AD (2013). Targeting the renin-angiotensin-aldosterone system in heart failure. Nat Rev Cardiol.

[5] Koitabashi N, Kass DA. (2012) Reverse remodeling in heart failure[mdash]mechanisms and therapeutic opportunities. Nat Rev Cardiol 9:147–157 10.1038/nrcardio.2011.172 22143079

[6] Zucker IH, Xiao L, Haack KK (2014). The central renin-angiotensin system and sympathetic nerve activity in chronic heart failure. Clin Sci (Lond).

[7] Cohn JN (2014). Heart failure in 2013: continue what we are doing to treat HF, but do it better. Nat Rev Cardiol.

[8] Wolfram JA, Donahue JK (2013). Gene therapy to treat cardiovascular disease. J Am Heart Assoc.

[9] Menasche P (2011). Cardiac cell therapy: lessons from clinical trials. J Mol Cell Cardiol.

[10] Fukushima S, Sawa Y, Suzuki K (2013). Choice of cell-delivery route for successful cell transplantation therapy for the heart. Future Cardiol.

[11] Behfar A, Crespo-Diaz R, Terzic A, Gersh BJ (2014). Cell therapy for cardiac repair; lessons from clinical trials. Nat Rev Cardiol.

[12] Sanganalmath SK, Bolli R (2013). Cell therapy for heart failure: a comprehensive overview of experimental and clinical studies, current challenges, and future directions. Circ Res.

[13] Tous E, Purcell B, Ifkovits J, Burdick J (2011). Injectable acellular hydrogels for cardiac repair. J Cardiovasc Transl Res.

[14] Chawengsub Y, Gauthier KM, Campbell WB (2009). Role of arachidonic acid lipoxygenase metabolites in the regulation of vascular tone. Am J Physiol Heart Circ Physiol.

[15] Dorris SL, Peebles RS (2012). PGI2 as a regulator of inflammatory diseases. Mediators Inflamm.

[16] Uchida T, Hazekawa M, Yoshida M, Matsumoto K, Sakai Y (2013). A novel long-acting prostacyclin agonist (ONO-1301) with an angiogenic effect: promoting synthesis of hepatocyte growth factor and increasing cyclic AMP concentration via IP-receptor signaling. J Pharmacol Sci.

[17] Takahashi HK, Iwagaki H, Tamura R (2003). Unique regulation profile of prostaglandin E1 on adhesion molecule expression and cytokine production in human peripheral blood mononuclear cells. J Pharmacol Exp Ther.

[18] Takahashi HK, Iwagaki H, Tamura R (2005). Differential effect of prostaglandins E1 and E2 on lipopolysaccharide-induced adhesion molecule expression on human monocytes. Eur J Pharmacol.

[19] Takahashi HK, Iwagaki H, Mori S, Yoshino T, Tanaka N, Nishibori M (2005). Prostaglandins E1 and E2 inhibit lipopolysaccharide-induced interleukin-18 production in monocytes. Eur J Pharmacol.

[20] Imanishi Y, Miyagawa S, Fukushima S (2013). Sustained-release delivery of prostacyclin analogue enhances bone marrow-cell recruitment and yields functional benefits for acute myocardial infarction in mice. PLoS One.

[21] Ishimaru K, Miyagawa S, Fukushima S (2013). Synthetic prostacyclin agonist, ONO1301, enhances endogenous myocardial repair in a hamster model of dilated cardiomyopathy: a promising regenerative therapy for the failing heart. J Thorac Cardiovasc Surg.

[22] Kubota Y, Miyagawa S, Fukushima S (2014). Impact of cardiac support device combined with slow-release prostacyclin agonist in a canine ischemic cardiomyopathy model. J Thorac Cardiovasc Surg.

[23] Shirasaka T, Miyagawa S, Fukushima S (2013). A slow-releasing form of prostacyclin agonist (ONO1301SR) enhances endogenous secretion of multiple cardiotherapeutic cytokines and improves cardiac function in a rapid-pacing–induced model of canine heart failure. J Thorac Cardiovasc Surg.

[24] Imawaka H, Sugiyama Y (1998). Kinetic study of the hepatobiliary transport of a new prostaglandin receptor agonist. J Pharmacol Exp Ther.

[25] Uchida T, Hazekawa M, Morisaki T, Yoshida M, Sakai Y (2013). Effect of antioxidants on the stability of ONO-1301, a novel long-acting prostacyclin agonist, loaded in PLGA microspheres. J Microencapsul.

[26] Tsai MK, Hsieh CC, Kuo HF (2014). Effect of prostaglandin I2 analogs on macrophage inflammatory protein 1α in human monocytes via I prostanoid receptor and cyclic adenosine monophosphate. J Invest Med.

[27] Nakamura K, Sata M, Iwata H (2007). A synthetic small molecule, ONO-1301, enhances endogenous growth factor expression and augments angiogenesis in the ischaemic heart. Clin Sci (Lond).

[28] Xiao CY, Yuhki KI, Hara A (2004). Prostaglandin E2 protects the heart from ischemia-reperfusion injury via its receptor subtype EP4. Circulation.

[29] Tanaka M, Sakai A, Uchida S (2004). Prostaglandin E2 receptor (EP4) selective agonist (ONO-4819.CD) accelerates bone repair of femoral cortex after drill-hole injury associated with local upregulation of bone turnover in mature rats. Bone.

[30] Sasaoka R, Terai H, Toyoda H, Imai Y, Sugama R, Takaoka K (2004). A prostanoid receptor EP4 agonist enhances ectopic bone formation induced by recombinant human bone morphogenetic protein-2. Biochem Biophys Res Commun.

[31] Pagkalos J, Leonidou A, As-Sultany M, Heliotis M, Mantalaris A, Tsiridis E (2012). Prostaglandin E(2) receptors as potential bone anabolic targets—selective EP4 receptor agonists. Curr Mol Pharmacol.

[32] Ninomiya T, Hosoya A, Hiraga T (2011). Prostaglandin E(2) receptor EP(4)-selective agonist (ONO-4819) increases bone formation by modulating mesenchymal cell differentiation. Eur J Pharmacol.

[33] Namikawa T, Terai H, Hoshino M (2007). Enhancing effects of a prostaglandin EP4 receptor agonist on recombinant human bone morphogenetic protein-2 mediated spine fusion in a rabbit model. Spine (Phila Pa 1976).

[34] Nakagawa K, Imai Y, Ohta Y, Takaoka K (2007). Prostaglandin E2 EP4 agonist (ONO-4819) accelerates BMP-induced osteoblastic differentiation. Bone.

[35] Marui A, Hirose K, Maruyama T (2006). Prostaglandin E2 EP4 receptor-selective agonist facilitates sternal healing after harvesting bilateral internal thoracic arteries in diabetic rats. J Thorac Cardiovasc Surg.

[36] Ito M, Nakayama K, Konaka A, Sakata K, Ikeda K, Maruyama T (2006). Effects of a prostaglandin EP4 agonist, ONO-4819, and risedronate on trabecular microstructure and bone strength in mature ovariectomized rats. Bone.

[37] Hayashi K, Fotovati A, Ali SA, Oda K, Oida H, Naito M (2005). Prostaglandin EP4 receptor agonist augments fixation of hydroxyapatite-coated implants in a rat model of osteoporosis. J Bone Joint Surg Br.

[38] Hagino H, Kuraoka M, Kameyama Y, Okano T, Teshima R (2005). Effect of a selective agonist for prostaglandin E receptor subtype EP4 (ONO-4819) on the cortical bone response to mechanical loading. Bone.

[39] Chang F, Mishima H, Ishii T (2007). Stimulation of EP4 receptor enhanced bone consolidation during distraction osteogenesis. J Orthop Res.

[40] Sekiguchi M, Konno SI, Kikuchi SI (2006). Effects on improvement of blood flow in the chronically compressed cauda equina: comparison between a selective prostaglandin E receptor (EP4) agonist and a prostaglandin E1 derivate. Spine.

[41] Kasai K, Sato SI, Suzuki K (2001). A novel prostaglandin E receptor subtype agonist, ONO-4819, attenuates acute experimental liver injury in rats. Hepatol Res.

[42] Honma Y, Arai I, Hashimoto Y (2005). Prostaglandin D2 and prostaglandin E2 accelerate the recovery of cutaneous barrier disruption induced by mechanical scratching in mice. Eur J Pharmacol.

[43] Nakagawa N, Yuhki KI, Kawabe JI (2012). The intrinsic prostaglandin E2-EP4 system of the renal tubular epithelium limits the development of tubulointerstitial fibrosis in mice. Kidney Int.

[44] Nakase H, Fujiyama Y, Oshitani N (2010). Effect of EP4 agonist (ONO-4819CD) for patients with mild to moderate ulcerative colitis refractory to 5-aminosalicylates: a randomized phase II, placebo-controlled trial. Inflamm Bowel Dis.

[45] Hishikari K, Suzuki JI, Ogawa M (2009). Pharmacological activation of the prostaglandin E2 receptor EP4 improves cardiac function after myocardial ischaemia/reperfusion injury. Cardiovasc Res.

[46] Ogawa M, Suzuki JI, Kosuge H, Takayama K, Nagai R, Isobe M (2009). The mechanism of anti-inflammatory effects of prostaglandin E2 receptor 4 activation in murine cardiac transplantation. Transplantation.

[47] Ngoc PB, Suzuki J, Ogawa M (2011). The anti-inflammatory mechanism of prostaglandin e2 receptor 4 activation in rat experimental autoimmune myocarditis. J Cardiovasc Pharmacol.

[48] O’Farrell FM, Attwell D (2014). A role for pericytes in coronary no-reflow. Nat Rev Cardiol.

[49] Dominguez-Rodriguez A, Abreu-Gonzalez P, Reiter RJ (2014). Cardioprotection and pharmacological therapies in acute myocardial infarction: challenges in the current era. World J Cardiol.

[50] Frangogiannis NG (2014). The inflammatory response in myocardial injury, repair, and remodelling. Nat Rev Cardiol.

[51] Camici PG, d’Amati G, Rimoldi O. (2015) Coronary microvascular dysfunction: mechanisms and functional assessment. Nat Rev Cardiol 12:48-62 10.1038/nrcardio.2014.160 25311229

[52] Giblett JP, West NEJ, Hoole SP (2014). Cardioprotection for percutaneous coronary intervention-Reperfusion quality as well as quantity. Int J Cardiol.

[53] Sivaraman V, Yellon DM (2014). Pharmacologic therapy that simulates conditioning for cardiac ischemic/reperfusion injury. J Cardiovasc Pharmacol Ther.

[54] Moukarbel GV, Ayoub CM, Abchee AB (2004). Pharmacological therapy for myocardial reperfusion injury. Curr Opin Pharmacol.

[55] Kloner RA, Forman MB, Gibbons RJ, Ross AM, Alexander RW, Stone GW (2006). Impact of time to therapy and reperfusion modality on the efficacy of adenosine in acute myocardial infarction: the AMISTAD-2 trial. Eur Heart J.

[56] Wang X, Wei M, Kuukasjärvi P (2009). The anti-inflammatory effect of bradykinin preconditioning in coronary artery bypass grafting (bradykinin and preconditioning). Scand Cardiovasc J.

[57] Wong GTC, Huang Z, Ji S, Irwin MG (2010). Remifentanil reduces the release of biochemical markers of myocardial damage after coronary artery bypass surgery: a randomized trial. J Cardiothorac Vasc Anesth.

[58] Lønborg J, Vejlstrup N, Kelbæk H (2012). Exenatide reduces reperfusion injury in patients with ST-segment elevation myocardial infarction. Eur Heart J.

[59] Kitakaze M, Asakura M, Kim J (2007). Human atrial natriuretic peptide and nicorandil as adjuncts to reperfusion treatment for acute myocardial infarction (J-WIND): two randomised trials. Lancet.

[60] Najjar SS, Rao SV, Melloni C (2011). Intravenous erythropoietin in patients with ST-segment elevation myocardial infarction: REVEAL: a randomized controlled trial. JAMA.

[61] Diaz R, Goyal A, Mehta SR (2007). Glucose-insulin-potassium therapy in patients with ST-segment elevation myocardial infarction. JAMA.

[62] Garcia C, Julier K, Bestmann L (2005). Preconditioning with sevoflurane decreases PECAM-1 expression and improves one-year cardiovascular outcome in coronary artery bypass graft surgery. Br J Anaesth.

[63] Yusuf S, Macmahon S, Collins R, Peto R (1998). Effect of intravenous nitrates on mortality in acute myocardial infarction: an overview of the randomised trials. Lancet.

[64] Kim JS, Kim J, Choi D (2010). Efficacy of high-dose atorvastatin loading before primary percutaneous coronary intervention in ST-segment elevation myocardial infarction. The STATIN STEMI trial. JACC Cardiovasc Interv.

[65] Mochly-Rosen D, Das K, Grimes KV (2012). Protein kinase C, an elusive therapeutic target?. Nat Rev Drug Discov.

[66] Piot C, Croisille P, Staat P (2008). Effect of cyclosporine on reperfusion injury in acute myocardial infarction. N Engl J Med.

[67] Schwartz BG, Levine LA, Comstock G, Stecher VJ, Kloner RA (2012). Cardiac uses of phosphodiesterase-5 inhibitors. J Am Coll Cardiol.

[68] Fukushima S, Coppen SR, Varela-Carver A (2006). Enhanced efficiency of superoxide dismutase-induced cardioprotection by retrograde intracoronary administration. Cardiovasc Res.

[69] Fukushima S, Coppen SR, Varela-Carver A (2006). A novel strategy for myocardial protection by combined antibody therapy inhibiting both P-selectin and intercellular adhesion molecule-1 via retrograde intracoronary route. Circulation.

[70] Hirata Y, Shimabukuro M, Uematsu E (2012). A synthetic prostacyclin agonist with thromboxane synthase inhibitory activity, ONO-1301, protects myocardium from ischemia/reperfusion injury. Eur J Pharmacol.

[71] Iwata H, Nakamura K, Sumi M (2009). Local delivery of synthetic prostacycline agonist augments collateral growth and improves cardiac function in a swine chronic cardiac ischemia model. Life Sci.

[72] Hirata Y, Soeki T, Akaike M, Sakai Y, Igarashi T, Sata M (2009). Synthetic prostacycline agonist, ONO-1301, ameliorates left ventricular dysfunction and cardiac fibrosis in cardiomyopathic hamsters. Biomed Pharmacother.

[73] Suzuki JI, Ogawa M, Sakai Y, Hirata Y, Isobe M, Nagai R (2012). A prostacycline analog prevents chronic myocardial remodeling in murine cardiac allografts. Int Heart J.

[74] Hirata Y, Kurobe H, Uematsu E (2013). Beneficial effect of a synthetic prostacyclin agonist, ONO-1301, in rat autoimmune myocarditis model. Eur J Pharmacol.

[75] Kainuma S, Miyagawa S, Fukushima S (2015). Cell-sheet therapy with omentopexy promotes arteriogenesis and improves coronary circulation physiology in failing heart. Mol Ther.

[76] Mann DL, Kubo SH, Sabbah HN (2012). Beneficial effects of the CorCap cardiac support device: five-year results from the Acorn Trial. J Thorac Cardiovasc Surg.

[77] Fukushima S, Campbell NG, Coppen SR (2011). Quantitative assessment of initial retention of bone marrow mononuclear cells injected into the coronary arteries. J Heart Lung Transplant.

[78] Fukushima S, Varela-Carver A, Coppen SR (2007). Direct intramyocardial but not intracoronary injection of bone marrow cells induces ventricular arrhythmias in a rat chronic ischemic heart failure model. Circulation.

[79] Miyagawa S, Saito A, Sakaguchi T (2010). Impaired myocardium regeneration with skeletal cell sheets—a preclinical trial for tissue-engineered regeneration therapy. Transplantation.

[80] Shudo Y, Miyagawa S, Fukushima S (2011). Novel regenerative therapy using cell-sheet covered with omentum flap delivers a huge number of cells in a porcine myocardial infarction model. J Thorac Cardiovasc Surg.

[81] Zamanian RT, Kudelko KT, Sung YK, de Jesus Perez V, Liu J, Spiekerkoetter E (2014). Current clinical management of pulmonary arterial hypertension. Circ Res.

[82] Ruan CH, Dixon RA, Willerson JT, Ruan KH (2010). Prostacyclin therapy for pulmonary arterial hypertension. Tex Heart Inst J.

[83] Kataoka M, Nagaya N, Satoh T (2005). A long-acting prostacyclin agonist with thromboxane inhibitory activity for pulmonary hypertension. Am J Respir Crit Care Med.

[84] Antoniu SA (2006). Non-prostanoid prostacyclin agonists for the treatment of pulmonary arterial hypertension. Expert Opin Investig Drugs.

[85] Obata H, Sakai Y, Ohnishi S (2008). Single injection of a sustained-release prostacyclin analog improves pulmonary hypertension in rats. Am J Respir Crit Care Med.

[86] Nakamura A, Nagaya N, Obata H (2013). Oral administration of a novel long-acting prostacyclin agonist with thromboxane synthase inhibitory activity for pulmonary arterial hypertension. Circ J.

[87] Murakami S, Nagaya N, Itoh T (2006). Prostacyclin agonist with thromboxane synthase inhibitory activity (ONO-1301) attenuates bleomycin-induced pulmonary fibrosis in mice. Am J Physiol Lung Cell Mol Physiol.

[88] Hayashi M, Koya T, Kawakami H (2010). A prostacyclin agonist with thromboxane inhibitory activity for airway allergic inflammation in mice. Clin Exp Allergy.

[89] Kimura Y, Koya T, Kagamu H (2013). A single injection of a sustained-release prostacyclin analog (ONO-1301MS) suppresses airway inflammation and remodeling in a chronic house dust mite-induced asthma model. Eur J Pharmacol.

[90] Hayashi K, Nagamatsu T, Oka T, Suzuki Y (1997). Modulation of anti-glomerular basement membrane nephritis in rats by ONO-1301, a non-prostanoid prostaglandin I2 mimetic compound with inhibitory activity against thromboxane A2 synthase. Jpn J Pharmacol.

[91] Yamasaki H, Maeshima Y, Nasu T (2011). Intermittent administration of a sustained-release prostacyclin analog ONO-1301 ameliorates renal alterations in a rat type 1 diabetes model. Prostaglandins Leukot Essent Fatty Acids.

[92] Nasu T, Kinomura M, Tanabe K (2012). Sustained-release prostacyclin analog ONO-1301 ameliorates tubulointerstitial alterations in a mouse obstructive nephropathy model. Am J Physiol Renal Physiol.

[93] Hazekawa M, Sakai Y, Yoshida M, Haraguchi T, Uchida T (2012). The effect of treatment with a sustained-release prostacyclin analogue (ONO-1301-loaded PLGA microsphere) on short-term memory impairment in rats with transient global cerebral ischemia. J Microencapsul.

[94] Hazekawa M, Sakai Y, Yoshida M, Haraguchi T, Uchida T (2012). Single injection of ONO-1301-loaded PLGA microspheres directly after ischaemia reduces ischaemic damage in rats subjected to middle cerebral artery occlusion. J Pharm Pharmacol.

[95] Xu Q, Nakayama M, Suzuki Y (2012). Suppression of acute hepatic injury by a synthetic prostacyclin agonist through hepatocyte growth factor expression. Am J Physiol Gastrointest Liver Physiol.

[96] Xu Q, Sakai K, Suzuki Y, Tambo C, Sakai Y, Matsumoto K (2013). Suppression of fibrogenic gene expression and liver fibrosis using a synthetic prostacyclin agonist. Biomed Res.

[97] Niina Y, Ito T, Oono T (2014). A sustained prostacyclin analog, ONO-1301, attenuates pancreatic fibrosis in experimental chronic pancreatitis induced by dibutyltin dichloride in rats. Pancreatology.

[98] Lederle FA, Freischlag JA, Kyriakides TC (2012). Long-term comparison of endovascular and open repair of abdominal aortic aneurysm. N Engl J Med.

[99] Malina M, Brunkwall J, Ivancev K, Jönsson J, Malina J, Lindblad B (2000). Endovascular healing is inadequate for fixation of dacron stent-grafts in human aortoiliac vessels. Eur J Vasc Endovasc Surg.

[100] Watanabe Y, Miyagawa S, Fukushima S (2014). Development of a prostacyclin-agonist–eluting aortic stent graft enhancing biological attachment to the aortic wall. J Thorac Cardiovasc Surg.

[101] Agarwal S, Tuzcu EM, Krishnaswamy A (2015). Transcatheter aortic valve replacement: current perspectives and future implications. Heart.

